# Are Current *Aspergillus sojae* Strains Originated from a Native Aflatoxigenic *Aspergillus* Species Population Also Present in California?

**DOI:** 10.1080/12298093.2023.2217495

**Published:** 2023-06-08

**Authors:** Perng-Kuang Chang, Sui Sheng T. Hua

**Affiliations:** a Southern Regional Research Center, Agricultural Research Service, U.S. Department of Agriculture, New Orleans, LA, USA; b Western Regional Research Center, Agricultural Research Service, U.S. Department of Agriculture, Albany, CA, USA

**Keywords:** *Aspergillus sojae*, *Aspergillus parasiticus*, single nucleotide polymorphism, aflatoxin gene cluster, cyclopiazonic acid, phylogeny

## Abstract

*Aspergillus sojae* has long been considered a domesticated strain of *Aspergillus parasiticus*. This study delineated relationships among the two species and an *Aspergillus* PWE36 isolate. Of 25 examined clustered aflatoxin genes of PWE36, 20 gene sequences were identical to those of *A. sojae*, but all had variations to those of *A. parasiticus*. Additionally, PWE36 developmental genes of conidiation and sclerotial formation, overall, shared higher degrees of nucleotide sequence identity with *A. sojae* genes than with *A. parasiticus* genes. Examination of defective cyclopiazonic acid gene clusters revealed that the PWE36 deletion pattern was identical only to those of *A. sojae*. Using *A. sojae* SMF134 genome sequence as a reference, visualization of locally collinear blocks indicated that PWE36 shared higher genome sequence homologies with *A. sojae* than with *A. parasiticus*. Phylogenetic inference based on genome-wide single nucleotide polymorphisms (SNPs) and total SNP counts showed that *A. sojae* strains formed a monophyletic clade and were clonal. Two (Argentinian and Ugandan) *A. parasiticus* isolates but not including an Ethiopian isolate formed a monophyletic clade, which showed that *A. parasiticus* population is genetically diverse and distant to *A. sojae*. PWE36 and *A. sojae* shared a most recent common ancestor (MRCA). The estimated divergence time for PWE36 and *A. sojae* was about 0.4 mya. Unlike *Aspergillus oryzae*, another koji mold that includes genetically diverse populations, the findings that current *A. sojae* strains formed a monophyletic group and shared the MRCA with PWE36 allow *A. sojae* to be continuously treated as a species for food safety reasons.

## 1. Introduction

*Aspergillus sojae*, like *Aspergillus oryzae*, is a highly valued koji (starter) mold used to produce fermented foods, such as miso and soy sauce [1]. It together with *Aspergillus parasiticus*, *A. oryzae*, and *Aspergillus flavus* is part of the genus’ section *Flavi* (“section” is a taxonomic rank in-between genus and species) [2]. Non-aflatoxigenic *A. sojae* is commonly believed to be a domesticated *A. parasiticus* strain. This is because, unlike *A. oryzae*, it has only been isolated from koji [3]. *A. parasiticus*, which produces aflatoxins B_1,_ B_2_, G_1_ and G_2_, and *A. sojae* are morphologically similar although they can be differentiated by conidial diameter and colony color [4]. These phenotypic differences, however, are subtle and intergradation often exists between them. Kurtzman et al. reported that DNA homology between *A. sojae* and *A. parasiticus* was 91%, and it was 100% between *A. oryzae* and *A. flavus*. Hence, they proposed that *A. sojae*, *A. parasiticus*, and *A. oryzae* should be reduced to varietal status [5]. One school of thought also holds that traits gained during domestication caused the koji molds to lose morphological and physiological characteristics that at present still persist in wild-type *A. parasiticus* and *A. flavus*.

Several atypical *A. parasiticus* isolates that produced B- and G-type aflatoxins were collected from pistachio nut fruits and designated as PWE (for Pistachio Winter Experiment) strains [6]. However, distinct colony morphological variations exist among them. Their morphologies also exhibit similarity and dissimilarity to those of *A. parasiticus* and *A. sojae* reference strains. The PWE isolates at the molecular level have a unique HA-coding insertion (CTCATG) in the aflatoxin pathway regulatory gene, *aflR*, which is identical to what has been known for *A. sojae* isolates [7]. This insertion is not present in *A. parasiticus aflR*. How close the PWE isolates are to *A. sojae* and to *A. parasiticus* was unclear. The presence or absence of the CTCATG sequence and nucleotides at position #1154 in *aflR* are sufficient for *Aspergillus* species identification. For example, these molecular features correctly reclassified non-aflatoxigenic *A. oryzae* ATCC18895 to *A. sojae* [8].

In the genomics era, high-throughput whole genome sequencing has become a routine. Genome sequence information for a fungal isolate now can be readily obtained in days. This has made molecular classification techniques employed in the past; for example, DNA complementarity, electrophoretic karyotypes, restriction fragment length polymorphism (RFLP), and random amplified polymorphic DNA (RAPD) become obsolete. Accurate identification of fungal species no longer relies on nucleotide sequences of polymerase chain reaction (PCR) fragments of coding or rRNA gene(s) [9]. Additionally, culture characteristics ancillary to the sequence techniques are affected by environmental and nutritional conditions, which makes it difficult to score morphological data without error. Misclassification of fungal isolates therefore is not uncommon [8,10].

In the present study, we sequenced the genome of a representative PWE36 strain. We first compared PWE36 genes, which included 25 clustered genes involved in aflatoxin biosynthesis and 23 key developmental genes associated with conidiation (13 genes) and sclerotial formation (10 genes), to the orthologous genes of *A. sojae* and *A. parasiticus*. We also explored their genetic relatedness based on deletion patterns in the cyclopiazonic acid (CPA) gene clusters. We further provided visualization of whole genome homologies using the well-assembled *A. sojae* SMF134 genome [11] as the reference. Lastly, we used genome-wide single nucleotide polymorphisms (SNPs) to assess the evolutionary origin of *A. sojae* and to delineate phylogenetic relationships among *A. sojae*, PWE36, and *A. parasiticus*.

## 2. Materials and methods

### 2.1. Fungal strains and assembled genome sequences

Table 1 lists *A. sojae* strains and *A. parasiticus* isolates, geographic locations of collection, and assembled genome sequences used in this study. The genome sequences were retrieved from the National Center for Biotechnology Information (NCBI) genome database (https://www.ncbi.nlm.nih.gov/genome/) with respective species names.

**Table 1.** *Aspergillus* genome sequences used in this study.

Species	Assembly	Size (Mb)	Origin	References
*A. parasiticus* SU-1	GCA_000956085.1	39.47	Uganda	Linz et al. [36]
*A. parasiticus* CBS117618	GCA_009176385.1	38.39	Argentina	Kjærbølling et al. [37]
*A. parasiticus* 68-5	GCA_001576805.1	30.14	Georgia, USA	Faustinelli et al. [31]
*A. parasiticus* E1319	GCA_013145845.1	38.94	Ethiopia	Arias et al. [9]
*A. parasiticus* E1337	GCA_013145875.1	39.15	Ethiopia	Arias et al. [9]
*A. parasiticus* E1348	GCA_013146005.1	39.35	Ethiopia	Arias et al. [9]
*A. parasiticus* E1443	GCA_013146145.1	41.45	Ethiopia	Arias et al. [9]
*Aspergillus* PWE36	GCA_019419805.1	38.50	California, USA	Hua et al. [6]; this study
*A. sojae* SMF134	GCA_008274985.1	40.11^a^	South Korea	Kim et al. [11]
*A. sojae* NBRC4239	GCA_000226655.2	39.67	Japan	Sato et al. [15]
*A. sojae* TK-83	GCA_009687765.1	40.04	Japan	Watarai et al. [29]
*A. sojae* TK-84	GCA_009687785.1	40.02	Japan	Watarai et al. [29]
*A. sojae* TK*-*85	GCA_009687805.1	40.05	Japan	Watarai et al. [29]

^a^The genome has been assembled at the chromosomal level. It contains eight chromosomes, and their GenBank accession numbers are CP035530.1 to CP035524.1.

### 2.2. Genome sequencing and assembly of *Aspergillus* PWE36

*Aspergillus* PWE36 is one of the several aflatoxigenic isolates collected at Wolfskill Grant Experimental Farm (University of California Davis, Winters, California, USA) [6]. Their colony morphology and conidial surface texture resemble to those of *A. sojae* and *A. parasiticus.* PWE36 was selected as the representative strain for genome sequencing. It was grown in yeast peptone dextrose (YPD) broth at 28 °C for 24 h. Harvested mycelia were frozen and grounded to a fine powder with a pestle and mortar. Fungal DNA was extracted using a MasterPure DNA Purification Kit (Epicentre^®^ Biotechnologies, Madison, Wisconsin, USA). Genome sequencing was completed through a service agreement with CosmosID (Germantown, Maryland, USA). The libraries were sequenced using a whole-genome shotgun approach on an Illumina MiSeq instrument. The genome coverage was 40×. Sequence reads were first quality controlled using BBDuk. Trimmed FASTA files were then subjected to reference guided metagenomic assembly using SPAdes [12]. A total of 1,434 contigs (N50 is 53,221) were assembled. The BioProject is PRJNA575261, and the genome sequence is under Accession number WELH00000000.1.

### 2.3. Sequence alignments of clustered aflatoxin genes and developmental genes

Twenty-five genes in the aflatoxin gene cluster of *A. parasiticus* SU-1 [13] served as the original sequence search templates (Table 2). Its GenBank accession number is AY371490. BlastN search against PWE36 genome assembly, GCA_019419805.1, was carried out first and corresponding gene sequences were obtained. The PWE36 gene sequences were then used in BlastN search against the assembled genome sequences of *A. parasiticus* and *A. sojae* (Table 1). Similarly, selected developmental genes related to conidiation and sclerotial production were first identified from *A. flavus* NRRL3357 (Table 3) and served as BlastN search query sequences against PWE36 genome assembly. The obtained PWE36 gene sequences were then used in BlastN search against the assembled genome sequences of *A. parasiticus* and *A. sojae*.

**Table 2.** Degrees of sequence identity of PWE36 aflatoxin biosynthesis genes to those of *Aspergillus sojae* strains and *Aspergillus parasiticus* isolates.

		*A. sojae*			*A. parasiticus* ^c^			
AF genes^a^	PWE36	NBRC4239	SMF134	TK strains (3)^b^	SU-1	CBS117618	68-5	Ethiopian isolates (4)^b^
*aflYa/nadA*	100.00	99.93	99.93	99.93	97.56	97.64	96.42	95.50, 95.26, 95.50, 96.60
*aflX/ordB*	100.00	100.00	100.00	100.00	99.25	99.38	97.13	92.24, 92.37, 92.49, 92.24
*aflW/moxY*	100.00	99.93	99.93	99.93	99.86	99.86	99.17	93.31, 93.25, 93.04, 92.83
*aflV/cypX*	100.00	100.00	100.00	100.00	99.51	99.64	98.12	94.24, 94.24, 94.30, 94.06
*aflK/vbs*	100.00	99.95	99.95	99.95	99.45	99.40	97.63	98.89, 98.89, 98.89, 98.69
*aflQ/ordA*	100.00	100.00	100.00	100.00	99.43	99.49	98.20	97.53, 97.53, 97.69, 99.49
*aflP/omtA*	100.00	100.00	100.00	100.00	99.26	99.26	96.98	96.37, 96.37, 96.30, 96.17
*aflO/omtB*	100.00	100.00	100.00	100.00	99.40	99.33	97.68	95.36, 95.36, 95.36, 95.21
*aflI/avfA*	100.00	100.00	100.00	100.00	98.83	98.60	98.49	91.38, 91.26, 91.38, 90.91
*aflL/verB*	100.00	100.00	100.00	100.00	99.42	99.55	98.52	93.83, 93.83, 93.64, 94.22
*aflG/avnA*	100.00	100.00	100.00	100.00	99.63	99.50	98.75	94.05, 93.88, 93.82, 94.24
*aflN/verA*	100.00	100.00	100.00	100.00	99.35	99.28	98.50	93.27, 93.08, 93.08, 93.27
*aflM/ver-1*	100.00	100.00	100.00	100.00	99.56	99.67	99.67	96.55, 96.66, 98.44, 96.55
*aflE/norA*	100.00	100.00	100.00	100.00	98.83	99.14	98.60	98.13, 98.29, 98.13, 98.13
*aflJ/estA*	100.00	100.00	100.00	100.00	98.10	99.20	96.81	97.50, 97.70, 97.70, 97.50
*aflH/adhA*	100.00	100.00	100.00	100.00	98.57	98.57	97.97	98.33, 98.45, 98.21, 98.33
*aflS/aflJ*	100.00	100.00	100.00	100.00	99.38	99.59	98.96	99.04, 99.24, 99.10, 98.97
*aflR*	100.00	99.93	99.93	99.93	99.40	99.40	97.87	98.14, 98.21, 98.14, 98.14
*aflB/hexB*	100.00	100.00	100.00	100.00	99.49	99.45	98.62	99.25, 99.20, 99.28, 99.28
*aflA/hexA*	100.00	100.00	100.00	100.00	99.55	99.77	99.55	99.08, 99.12, 99.14, 99.14
*aflD/nor1*	100.00	100.00	100.00	100.00	99.39	99.49	99.29	99.80, 99.80, 99.80, 99.80
*aflC/pksA*	100.00	99.97	99.97	99.97	99.43	99.35	98.45	99.25, 99.36, 99.36, 99.38
*aflT*	100.00	100.00	100.00	100.00	99.84	99.89	98.37	98.89, 99.00, 99.00 99.31
*aflU/cypA*	100.00	100.00	100.00	100.00	99.77	99.55	97.45	96.31, 96.42, 99.21, 96.42
*aflF/norB*	100.00	100.00	100.00	100.00	99.74	99.74	99.13	98.61, 98.61, 98.35, 98.52

^a^Gene designations are from *A. parasiticus* SU-1 [13]. The GenBank accession number is AY371490.

^b^The three *A. sojae* TK strains (83, 84, and 85) have identical nucleotide sequences.

^c^CBS117618 was collected from the leaf of an Argentinian wild peanut species (*Arachis correntina*). SU-1 and 68-5 were isolated from peanut seeds from Uganda and Georgia, USA, respectively. The four *A. parasiticus* Ethiopian isolates are E1319, E1337, E1348, and E1443 (Table 1).

**Table 3.** Degrees of sequence identity of PWE36 developmental genes to those of *Aspergillus sojae* strains and *Aspergillus parasiticus* isolates.

		*A. sojae*		*A. parasiticus*		
Genes^a^	Gene products	NBRC4239	SMF134	SU-1	CBS117618	68-5
	Conidiation related					
*fluG*	Extracellular developmental signal protein	99.89	99.89	99.10	99.14	98.53
*flbA*	Developmental regulator	100.00	100.00	99.55	99.71	98.23
*flbB*	Basic-zipper-type transcription factor	100.00	100.00	99.72	99.72	98.41
*flbC*	C_2_H_2_ conidiation transcription factor	99.92	99.92	99.83	99.92	98.83
*flbD*	Conidiophore development protein	100.00	100.00	99.79	99.79	99.79
*flbE*	Conserved hypothetical protein	100.00	100.00	99.55	99.55	98.64
*fadA*	G-protein complex alpha subunit	99.76	99.76	99.68	99.68	97.90
*brlA*	C_2_H_2_ type conidiation transcription factor	99.92	99.92	99.45	99.45	98.66
*hymA*	Conidiophore development protein	99.93	99.93	99.93	99.86	98.75
*stuA*	APSES transcription factor	99.91	99.91	99.67	99.67	99.38
*medA*	Transcriptional regulator	99.84	99.84	99.79	99.79	98.64
*abaA*	Transcription factor	100.00	100.00	99.54	99.54	98.63
*wetA*	Developmental regulatory protein	99.82	99.82	99.59	99.65	98.58
	Sclerotial formation related					
*sclR*	HLH DNA-binding protein	100.00	100.00	98.23	98.34	98.44
*veA*	Developmental regulator	99.39	99.39	99.66	99.78	98.77
*velB*	Nucleoside diphosphatase	100.00	100.00	99.36	99.28	97.59
*velC*	Conserved protein	99.79	99.79	99.16	99.37	96.42
*vosA*	Developmental regulator	99.90	99.90	99.75	99.85	98.44
*nsdC*	C_2_H_2_ zinc finger protein	100.00	100.00	100.00	99.95	99.75
*nsdD*	Sexual development factor	100.00	100.00	99.74	99.68	98.41
*laeA*	Secondary metabolism regulator	99.78	99.78	99.89	100.00	99.66
*aswA*	C6-type transcription factor	100.00	100.00	99.81	99.81	99.16
*sfgA*	C6-type transcription factor	99.15	99.15	99.36	99.26	–

^a^Genes of *A. flavus* NRRL33357 were used to BlastN the PWE36 genome sequence. The aligned PWE36 sequences were retrieved and used to BlastN the genome sequences of *A. parasiticus* and *A. sojae*. Gene sequences of PWE36 are 100.00%.

### 2.4. Characterization of *pksA* genes and *aflR* genes of *A. parasiticus*, PWE36, and *A. sojae*

The polyketide synthase gene, *pksA/aflC*, and the aflatoxin pathway-specific regulatory gene, *aflR*, of *A. sojae* SRRC1123 (GenBank accession numbers: AY607769 and KT829484) were retrieved from the NCBI nucleotide database (https://www.ncbi.nlm.nih.gov/nuccore/?term=). The sequence portions containing the desired nucleotide substitutions and insertion/deletion were identified and used to align the assembled genome sequences of *A. parasiticus* and *A. sojae* (Table 1) with BlastN search.

### 2.5. Characterization of defective CPA gene clusters of *A. parasiticus*, PWE36, and *A. sojae*

Unlike *A. flavus* that produces CPA, *A. sojae* and *A. parasiticus* are unable to produce this metabolite because their CPA gene clusters are defective [14,15]. For determining deletion patterns of the CPA gene clusters in *A. sojae* and *A. parasiticus*, the 16.8 kb CPA gene cluster of *A. flavus* AF36 (GenBank accession number: JN712209) was used as the alignment template in BlastN search against genome sequences (Table 1). Localization and detailed analysis of deleted sequence regions were performed manually.

### 2.6. Visualization of genome sequence homology and phylogeny of *A. sojae*, PWE36, and *A. parasiticus*

Mauve, an online system for aligning multiple genome sequences was downloaded (http://darlinglab.org/mauve/mauve.html) [16]. Its program “progressiveMauve” was used to extract nucleotide variations that exist between compared genomes. For visual representation of two genome sequences aligned, the program “Move Contigs” of Mauve was used to draw locally collinear blocks (LCBs), which show homologous regions between compared genome assemblies. *A. sojae* SMF134 genome sequence, which has been assembled into eight chromosomes [11], was used as the reference. Phylogenetic inference with concatenated total SNP sequences was performed by the weighted Neighbor-Joining method of the online program MAFFT (version 7) (https://mafft.cbrc.jp/alignment/server/phylogeny.html) [17]. Aligning genome sequences, extracting SNPs of aligned paired or all genomes, filtering out noise (i.e., gaps and ambiguous bases), concatenating cleaned SNPs, and converting sequences to FASTA format were carried out using a custom JavaScript [18]. A phylogeny with rectangular tree layout was prepared using FigTree v1.4.3 (http://tree.bio.ed.ac.uk/software/figtree/).

## 3. Results and discussion

### 3.1. Comparison of aflatoxin biosynthesis genes and those associated with development

In contrast to *A. parasiticus*, which is aflatoxigenic, *A. sojae* does not produce aflatoxins. Although aflatoxins (B_1_, B_2_, G_1_ and G_2_) are not essential for growth and development of producing fungi, the gene cluster is believed to have been maintained for 25 million years [19], which suggests that producing these polyketide-derived metabolites in nature has an adaptive value. In this study, the comparison of 25 genes in the aflatoxin gene clusters showed that nucleotide sequences, which include exons and introns, of 20 genes between PWE36 and the five *A. sojae* strains were identical (Table 2). In contrast, all 25 genes between PWE36 and the *A. parasiticus* isolates collected from different geographical locations had minor sequence variations. Consistently, developmental genes of PWE36 associated with conidiation shared higher degrees of nucleotide sequence identity with *A. sojae* genes than with *A. parasiticus* genes. But three of the 10 PWE36 developmental genes associated with sclerotial formation, *veA* [20,21], *laeA* [22] and *sfgA* [23], had higher sequence variations when compared to *A. sojae* genes than to *A. parasiticus* genes. Thousands of years’ use of koji for preparation of East Asian traditional fermented foods has resulted in morphological changes of *A. oryzae* and *A. sojae*. For example, *A. oryzae* has sparse sporulation, floccose aerial mycelia and few or no sclerotia, and aging colonies change color toward brown rather than green as seen for *A. flavus* and *A. parasiticus* [24,25]. Floccose mycelia and aged color change also are reported for *A. sojae*. It is not known whether the selection pressure from the solid-state cultivation during fermentation processes, which enables fast growth and conidiation, forces the domesticated koji molds to adapt to specific production environments and affects formation of sclerotia, another type of propagules produced under unfavorable growth conditions.

### 3.2. SNPs in *pksA* genes and indels in *aflR* genes of *A. parasiticus*, PWE36, and *A. sojae*

**Figure 1.**
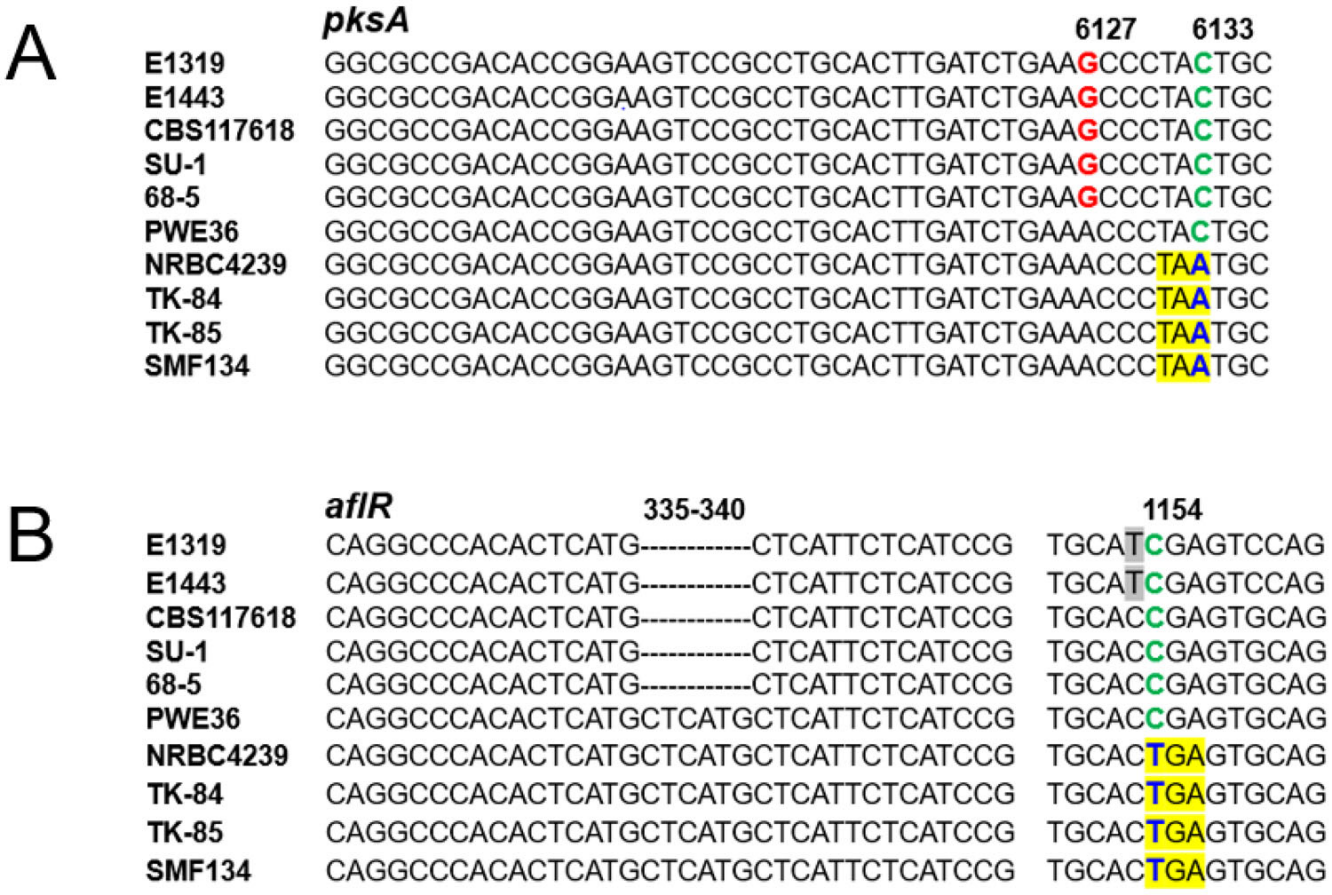
Alignment of portions of *pksA/aflC* sequences (A); *aflR* sequences (B) from *Aspergillus parasiticus* isolates, PWE36, and *Aspergillus sojae* strains. SNPs in blue that yield pre-termination codons in *pksA/aflC* (TAA) and *aflR* (TGA) genes, respectively, are yellow highlighted. Numbers correspond to nucleotide positions in *A. sojae* gene sequences.

Functional *pksA/aflC* and *aflR* genes are required for aflatoxin production in *A. parasiticus* [26,27]. Sequence comparison has indicated that both orthologous genes in *A. sojae* are defective [7,28], thereby rendering *A. sojae* unable to produce aflatoxins. In this study, the nucleotides of *pksA/aflC* genes at positions #6127 and #6133 for all *A. parasiticus* isolates and all *A. sojae* strains examined were “GC” and “AA”, respectively, which are consistent with previous findings (Figure 1(A)). Notably, PWE36 had intermediate nucleotide changes. At position #6127 it had “A” identical to that in *A. sojae*, but at position #6133 it had “C” identical to that in *A. parasiticus*. The acquisition of “A” in position #6133 in *A. sojae*, which results in a pre-termination codon, likely occurred after PWE36-like ancestor (and *A. sojae*) diverged from *A. parasiticus*. Probably the mutation was introduced during domestication. Similarly, the insertion (duplication) of the CTCATG nucleotides in *aflR* of PWE36 indicated its divergence from *A. parasiticus* (Figure 1(B)). The ensuring change of nucleotide “C” at position #1154 to “T” in *A. sojae* further suggests a genetic transition from a PWE36-like ancestor to *A. sojae.*

Historically speaking, use of koji in food fermentation began first in China over 2,000 to 3000 years ago, and the technology was adopted by other regions such as Japan and Korea during different periods [25,29]. To investigate whether PWE36-like *Aspergillus* exists in regions(s) other than California, we therefore searched the NCBI databases for possible candidates. To our surprise, *A. parasiticus* OPS651, isolated from peanut in China and producing B- and G-type aflatoxins [30], also contained the additional HA-coding sequence, CTCATG. It is probable that POS651 is genetically close to PWE36. As more field *Aspergillus* isolates having molecular features identical to PWE36 are collected and their genomes sequenced, we would have a better understanding of the phylogenetic relationship of *A. parasiticus* and *A. sojae*.

### 3.3. Defective CPA gene clusters of *A. parasiticus*, PWE36, and *A. sojae*

**Figure 2.**
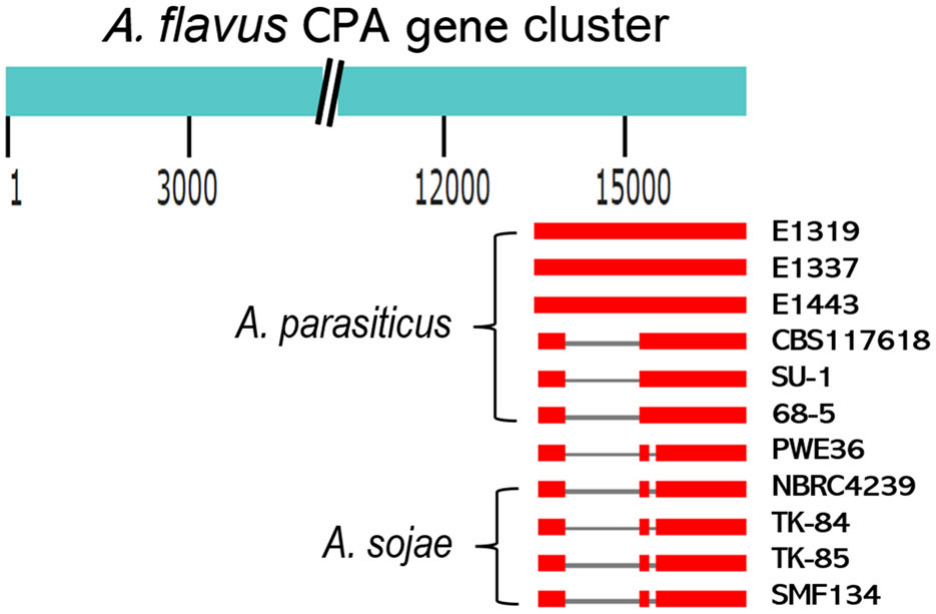
Schematic representations of deletions in the CPA gene clusters of *Aspergillus parasiticus* isolates, PWE36, and *Aspergillus sojae* strains. The CPA gene cluster of *Aspergillus flavus* AF36 (16.8 kb) was used as the alignment template. The comparisons on the lower panel were drawn to scale. Blank space indicates deleted portions. Grey lines are nonhomologous regions of replacement and the 112 bp deletions. Thick red lines are highly homologous sequences.

*A. parasiticus* and *A. sojae*, unlike *A. flavus* and *A. oryzae*, are unable to produce CPA because they do not have a complete CPA gene cluster, which includes at least three characterized genes, *maoA*, *dmaT* and *pks-nrps/cpaA* [3,14,15]. The exact deletion(s) in *A. parasiticus* and *A. sojae* CPA gene clusters had not yet been determined. To examine the relationship of PWE36 with *A. parasiticus* and with *A. sojae*, we defined deletion patterns in their CPA gene clusters. Using the complete *A. flavus* CPA gene cluster as a reference, we found that only about 3.5 kb in the 3′ portion that harbors the *pks-nrps/cpaA* gene was retained in the incomplete CPA gene clusters of the Ethiopian *A. parasiticus* isolates (Figure 2). Comparatively, *A. parasiticus* isolates CBS117618, SU-1, and 68-5 [31] had a further 1.2 kb deletion. On closer manual examination, we found that the corresponding 1.2 kb region in these isolates was replaced by a 7.7 kb nonhomologous region (data not shown). The CPA gene clusters of the four *A. sojae* strains and PWE36 had the same 7.7 kb replacement; all also had an additional short 112 bp deletion. The transition of these specific deletion patterns suggests that PWE36 is genetically closer to *A. sojae* than to *A. parasiticus*.

### 3.4. Visualization of genome-wide sequence homology

**Figure 3.**
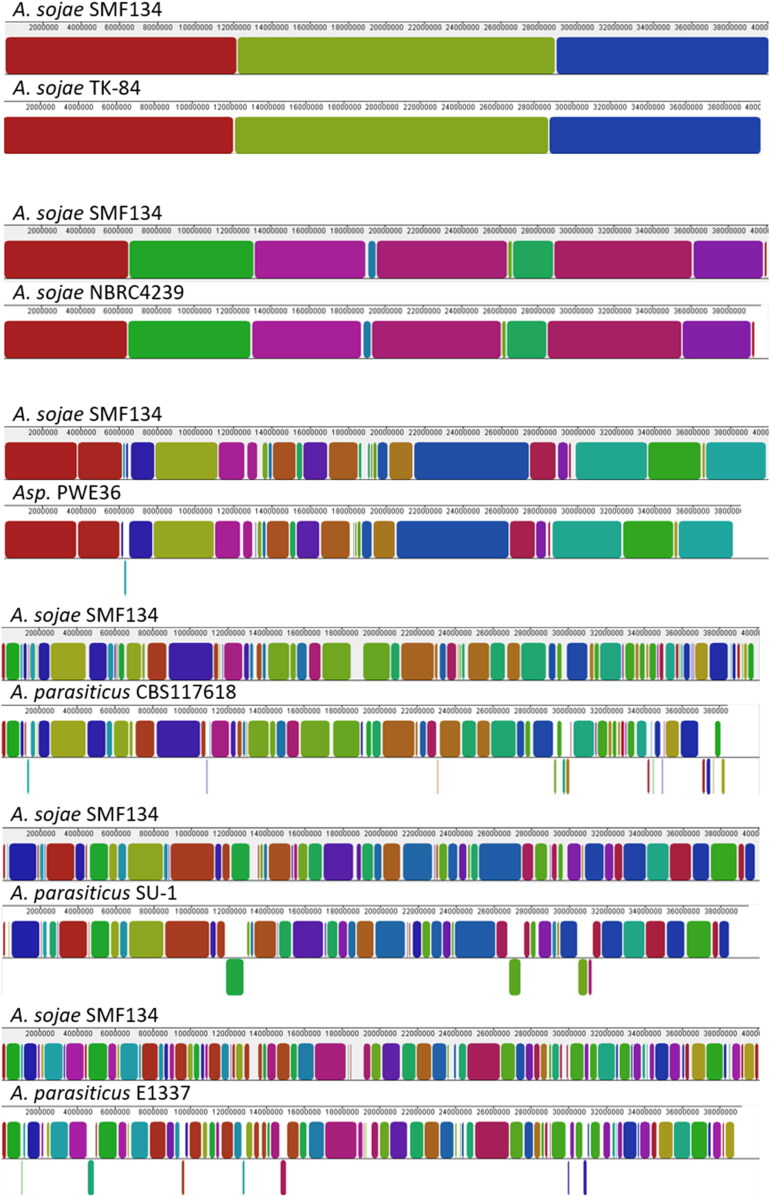
Schematic representation of homologous regions in genome sequences of *Aspergillus sojae* strains, PWE36, and *Aspergillus parasiticus* isolates. The genome sequence of *A. sojae* SMF134 that had been assembled at the chromosome level was used as the reference; it was concatenated in chromosome order (from I to VIII). Colored blocks are locally collinear blocks (LCBs), which indicate homologous regions between two genomes. The LCB weight was set at around 550 except for the SMF134/TK-84 pair, of which the lowest score was 24,283. Regions that were split and translocated in a compared genome are shown as inverted segments at the bottom.

To visualize sequence homologies among the genomes of *A. sojae*, PWE36, and *A. parasiticus*, we used the *A. sojae* SMF134 genome sequence as a reference, whose eight chromosomes had been assembled [11]. The alignment template was based on concatenated sequences of the chromosomes. In Figure 3, homologous genome sequence regions were indicated by colored collinear blocks (LCBs); the larger the LCBs present the wider the homologous regions are. As revealed, genome sequences of *A. sojae* SMF134, TK-84, and NBRC4329 were highly homologous. In contrast, the SMF134 genome sequence shared much smaller LCBs with those of the three *A. parasiticus* isolates. Additionally, we found that various sequence translocations were present in the *A. parasiticus* genomes. Overall, the PWE36 genome sequence shared higher degrees of homology with those of *A. sojae* strains than with the *A. parasiticus* isolates.

### 3.5. SNP counts of paired genome sequence alignments and total SNP-based phylogeny

**Figure 4.**
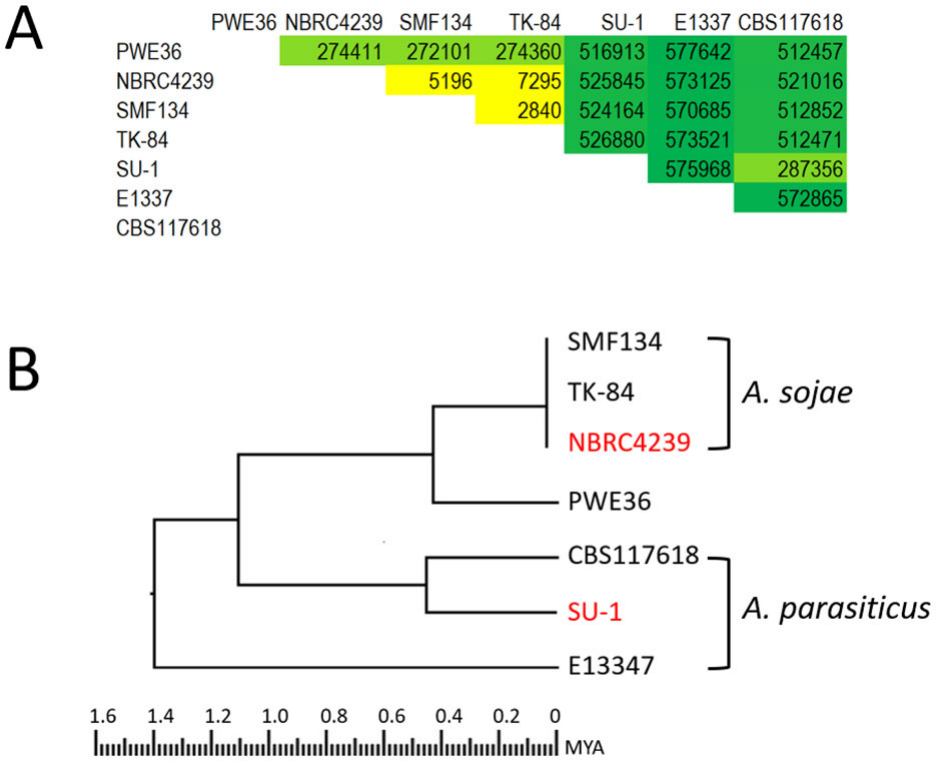
(A) Total SNP counts from paired genome sequence comparisons among PWE36, *Aspergillus sojae* strains, and *Aspergillus parasiticus* isolates; (B) Phylogenetic tree inferred from concatenated sequences of total SNPs by the Neighbor-Joining method with 100 bootstrap iterations. The number of total SNP count for each sequence was 618,048. The estimated divergence time (1.1 mya) between *A. parasiticus* SU-1 and *A. sojae* NRBC4239 was derived from the reference divergence time scale (3.8 mya) between *Aspergillus flavus* NRRL3357 and *Aspergillus oryzae* RIB40 [38].

In comparison to *A. oryzae*, of which over 200 genome sequences are publicly available, only a few *A. sojae* and less than 10 *A. parasiticus* genome sequences have been deposited to NCBI. Of the three *A. sojae* strains, SMF134 and TK-84 [11,29] shared the lowest total SNP count (2840) with each other (Figure 4(A)). NBRC4239 [15] also shared low total SNP counts with SM134 and TK-84, respectively. These low SNP counts suggest that current *A. sojae* strains are clonal. Low total SNP counts of *A. oryzae* clonal strains such as 3.042 and 100-8 (=1246) from China [32,33] as well as SRCM101975 and SRCM101989 from South Korea (=379) [34] also have been reported. Total SNP counts between the Ethiopian *A. parasiticus* isolate (E1337) and either CBS117618 or SU-1 were high although the count shared by CBS117618 and SU-1, collected respectively from the leaf of an Argentinian wild peanut species and a Ugandan peanut [35,36], was only about half. It is not clear whether geographic separation and niche adaptation have a bearing on the markedly different evolutionary distance. Alternately, E1337 might be a species very closely related to *A. parasiticus*. The total SNP counts among *A. parasiticus* isolates varied greatly (Figure 4(A)) and can be translated into approximately 98.6% to 99.3% genome sequence identity. Isolates of *A. flavus* (S- and L-morphotypes) and *A. oryzae* share overall total SNP counts of 220,000 and 300,000, respectively [34]. Hence, *A. parasiticus* has higher genetic diversity than *A. flavus* and *A. oryzae* (∼99.2% to 99.4% genome sequence identity) despite that a large group of *A. oryzae* has been shown to be phylogenetically closer to *A. aflatoxiformans* than to *A. flavus* [2,18,37]. PWE36 total SNP counts shared with the three *A. sojae* strains were in that range reported for *A. flavus* and *A. oryzae*. They were about four- to 10-fold of those shared among the *A. sojae* strains. The total SNP counts shared by PWE36 and the *A. sojae* strains were only half of those shared by PWE36 and the *A. parasiticus* isolates (Figure 4(A)), which indicates close genetic relatedness of the former aspergilli.

### 3.6. Divergence of current *A. sojae* strains from PWE36

A phylogenetic tree inferred by the Neighbor-Joining (NJ) method, using the concatenated sequences of total SNPs, was consistent with those inferred based on total SNP counts. For example, *A. sojae* SMF134, NBRC4239, and TK-84 comprised a monophyletic clade. *A. parasiticus* CBS117618 and SU-1 grouped in a separate monophyletic clade (Figure 4(B)). Notably, PWE36 shared a most recent common ancestor (MRCA) with the *A. sojae* strains and were phylogenetically distant from the three *A. parasiticus* isolates. A study inferring evolutionary relationships of *Aspergillus* species based on 1668 Benchmarking Universal Single-Copy Ortholog (BUSCO) genes has concluded that *A. parasiticus* (SU-1) and *A. sojae* (NRBC4239) diverged about 1.1 million years ago [38]. We used the phylogenetic tree derived from total SNP counts and extrapolated that current *A. sojae* strains diverged from *Aspergillus* PWE36 roughly 430 thousand years ago. PWE36 was much more distant from Ethiopian *A. parasiticus* isolates (about 1.4 million years ago) than from Ugandan and Argentinian *A. parasiticus* isolates. Nonetheless, PWE36 divergence from *A. parasiticus* occurred much more recent than the 3.8 million years ago estimated for *A. flavus* (NRRL3357) and *A. oryzae* (RIB40) [38].

## References

[1] Ito K, Matsuyama A. Koji molds for Japanese soy sauce brewing: characteristics and key enzymes. J Fungi. 2021;7(8):658. 10.3390/jof7080658 PMC8399179 34436196

[2] Frisvad JC, Hubka V, Ezekiel CN, et al. Taxonomy of *Aspergillus* section *flavi* and their production of aflatoxins, ochratoxins and other mycotoxins. Stud Mycol. 2019;93:1–63. 30108412 10.1016/j.simyco.2018.06.001 PMC6080641

[3] Kim KM, Lim J, Lee JJ, et al. Characterization of *Aspergillus sojae* isolated from Meju, Korean traditional fermented soybean brick. J Microbiol Biotechnol. 2017;27(2):251–261. 27880961 10.4014/jmb.1610.10013

[4] Klich MA. Identification of common *Aspergillus* species. Utrecht: Centraalbureau voor Schimmelcultures, 2002.

[5] Kurtzman CP, Smiley MJ, Robnett CJ, et al. DNA relatedness among wild and domesticated species in the *Aspergillus flavus* group. Mycologia. 1986;78(6):955–959.

[6] Hua SST, Parfitt DE, Sarreal SBL, et al. First report of an atypical new *Aspergillus parasiticus* isolates with nucleotide insertion in *aflR* gene resembling to *A. sojae*. Mycotoxin Res. 2018;34(2):151–157. 29464607 10.1007/s12550-018-0309-2

[7] Takahashi T, Chang P-K, Matsushima K, et al. Nonfunctionality of *Aspergillus sojae aflR* in a strain of *Aspergillus parasiticus* with a disrupted *aflR* gene. Appl Environ Microbiol. 2002;68(8):3737–3743. 12147467 10.1128/AEM.68.8.3737-3743.2002 PMC124037

[8] Watson AJ, Fuller LJ, Jeenes DJ, et al. Homologs of aflatoxin biosynthesis genes and sequence of *aflR* in *Aspergillus oryzae* and *Aspergillus sojae*. Appl Environ Microbiol. 1999;65(1):307–310. 9872797 10.1128/aem.65.1.307-310.1999 PMC91020

[9] Arias RS, Orner VA, Martinez-Castillo J, et al. *Aspergillus* section *flavi*, need for a robust taxonomy. Microbiol Resour Announc. 2021;10(48):e0078421. 34854700 10.1128/MRA.00784-21 PMC8638576

[10] Houbraken J, Visagie CM, Frisvad JC. Recommendations to prevent taxonomic misidentification of genome-sequenced fungal strains. Microbiol Resour Announc. 2021;10(48):e0107420. 34854710 10.1128/MRA.01074-20 PMC8638587

[11] Kim KU, Kim KM, Choi YH, et al. Whole genome analysis of *Aspergillus sojae* SMF 134 supports its merits as a starter for soybean fermentation. J Microbiol. 2019;57(10):874–883. 31250400 10.1007/s12275-019-9152-1

[12] Bankevich A, Nurk S, Antipov D, et al. SPAdes: a new genome assembly algorithm and its applications to single-cell sequencing. J Comput Biol. 2012;19(5):455–477. 22506599 10.1089/cmb.2012.0021 PMC3342519

[13] Yu J, Chang P-K, Ehrlich KC, et al. Clustered pathway genes in aflatoxin biosynthesis. Appl Environ Microbiol. 2004;70(3):1253–1262. 15006741 10.1128/AEM.70.3.1253-1262.2004 PMC368384

[14] Chang P-K, Horn BW, Dorner JW. Clustered genes involved in cyclopiazonic acid production are next to the aflatoxin biosynthesis gene cluster in *Aspergillus flavus*. Fungal Genet Biol. 2009;46(2):176–182. 19038354 10.1016/j.fgb.2008.11.002

[15] Sato A, Oshima K, Noguchi H, et al. Draft genome sequencing and comparative analysis of *Aspergillus sojae* NBRC4239. DNA Res. 2011;18(3):165–176. 21659486 10.1093/dnares/dsr009 PMC3111232

[16] Darling AC, Mau B, Blattner FR, et al. Mauve: multiple alignment of conserved genomic sequence with rearrangements. Genome Res. 2004;14(7):1394–1403. 15231754 10.1101/gr.2289704 PMC442156

[17] Katoh K, Misawa K, Kuma K, et al. MAFFT: a novel method for rapid multiple sequence alignment based on fast Fourier transform. Nucleic Acids Res. 2002;30(14):3059–3066. 12136088 10.1093/nar/gkf436 PMC135756

[18] Chang P-K, Chang TD, Katoh K. Deciphering the origin of *Aspergillus flavus* NRRL21882, the active biocontrol agent of Afla-Guard. Lett Appl Microbiol. 2021;72(5):509–516. 33251654 10.1111/lam.13433

[19] Ehrlich KC, Yu J, Cotty PJ. Aflatoxin biosynthesis gene clusters and flanking regions. J Appl Microbiol. 2005;99(3):518–527. 16108793 10.1111/j.1365-2672.2005.02637.x

[20] Calvo AM, Bok J, Brooks W, et al. *veA* is required for toxin and sclerotial production in *Aspergillus parasiticus*. Appl Environ Microbiol. 2004;70(8):4733–4739. 15294809 10.1128/AEM.70.8.4733-4739.2004 PMC492383

[21] Chang P-K, Scharfenstein LL, Li P, et al. *Aspergillus flavus* VelB acts distinctly from VeA in conidiation and may coordinate with FluG to modulate sclerotial production. Fungal Genet Biol. 2013;58–59:71–79. 10.1016/j.fgb.2013.08.009 23994319

[22] Kale SP, Milde L, Trapp MK, et al. Requirement of LaeA for secondary metabolism and sclerotial production in *Aspergillus flavus*. Fungal Genet Biol. 2008;45(10):1422–1429. 18667168 10.1016/j.fgb.2008.06.009 PMC2845523

[23] Yuan XY, Li JY, Zhi QQ, et al. SfgA renders *Aspergillus flavus* more stable to the external environment. J Fungi. 2022;8(6):638. 10.3390/jof8060638 PMC9224668 35736121

[24] Jorgensen TR. Identification and toxigenic potential of the industrially important fungi, *Aspergillus oryzae* and *Aspergillus sojae*. J Food Prot. 2007;70(12):2916–2972. 18095455 10.4315/0362-028x-70.12.2916

[25] Machida M, Yamada O, Gomi K. Genomics of *Aspergillus oryzae*: learning from the history of koji mold and exploration of its future. DNA Res. 2008;15(4):173–183. 18820080 10.1093/dnares/dsn020 PMC2575883

[26] Feng GH, Leonard TJ. Characterization of the polyketide synthase gene (*pksL1*) required for aflatoxin biosynthesis in *Aspergillus parasiticus*. J Bacteriol. 1995;177(21):6246–6254. 7592391 10.1128/jb.177.21.6246-6254.1995 PMC177466

[27] Chang P-K, Ehrlich KC, Yu J, et al. Increased expression of *Aspergillus parasiticus aflR*, encoding a sequence-specific DNA-binding protein, relieves nitrate inhibition of aflatoxin biosynthesis. Appl Environ Microbiol. 1995;61(6):2372–2377. 7793958 10.1128/aem.61.6.2372-2377.1995 PMC167509

[28] Chang P-K, Matsushima K, Takahashi T, et al. Understanding nonaflatoxigenicity of *Aspergillus sojae*: a windfall of aflatoxin biosynthesis research. Appl Microbiol Biotechnol. 2007;76(5):977–984. 17665189 10.1007/s00253-007-1116-4

[29] Watarai N, Yamamoto N, Sawada K, et al. Evolution of *Aspergillus oryzae* before and after domestication inferred by large-scale comparative genomic analysis. DNA Res. 2019;26(6):465–472. 31755931 10.1093/dnares/dsz024 PMC6993814

[30] Garber NP, Cotty PJ. *Aspergillus parasiticus* communities associated with sugarcane in the Rio Grande Valley of Texas: implications of global transport and host association within *Aspergillus* section *flavi*. Phytopathology. 2014;104(5):462–471. 24224872 10.1094/PHYTO-04-13-0108-R

[31] Faustinelli PC, Wang XM, Palencia ER, et al. Genome sequences of eight *Aspergillus flavus* spp. and one *A. parasiticus* sp., isolated from peanut seeds in Georgia. Genome Announc. 2016;4(2):e00278–e00216. 27081142 10.1128/genomeA.00278-16 PMC4832170

[32] Zhao G, Yao Y, Hou L, et al. Draft genome sequence of *Aspergillus oryzae* 100-8, an increased acid protease production strain. Genome Announc. 2014;2(3):e00548–14. 24903875 10.1128/genomeA.00548-14 PMC4047454

[33] Zhao G, Yao Y, Qi W, et al. Draft genome sequence of *Aspergillus oryzae* strain 3.042. Eukaryot Cell. 2012;11(9):1178. 22933657 10.1128/EC.00160-12 PMC3445974

[34] Chang P-K. Genome-wide nucleotide variation distinguishes *Aspergillus flavus* from *Aspergillus oryzae* and helps to reveal origins of atoxigenic *A. flavus* biocontrol strains. J Appl Microbiol. 2019;127(5):1511–1520. 31429498 10.1111/jam.14419

[35] Pildain MB, Frisvad JC, Vaamonde G, et al. Two novel aflatoxin-producing *Aspergillus* species from Argentinean peanuts. Int J Syst Evol Microbiol. 2008;58(3):725–735. 18319485 10.1099/ijs.0.65123-0

[36] Linz JE, Wee J, Roze LV. *Aspergillus parasiticus* SU-1 genome sequence, predicted chromosome structure, and comparative gene expression under aflatoxin-inducing conditions: evidence that differential expression contributes to species phenotype. Eukaryot Cell. 2014;13(8):1113–1123. 24951444 10.1128/EC.00108-14 PMC4135788

[37] Kjærbølling I, Vesth T, Frisvad JC, et al. A comparative genomics study of 23 *Aspergillus* species from section *flavi*. Nat Commun. 2020;11(1):1106. 32107379 10.1038/s41467-019-14051-y PMC7046712

[38] Steenwyk JL, Shen XX, Lind AL, et al. A robust phylogenomic time tree for biotechnologically and medically important fungi in the genera *Aspergillus* and *Penicillium*. mBio. 2019;10(4):e00925–19. 31289177 10.1128/mBio.00925-19 PMC6747717

